# Genotoxicity-suppressing effect of aqueous extract of *Connarus ruber* cortex on cigarette smoke-induced micronuclei in mouse peripheral erythrocytes

**DOI:** 10.1186/s41021-015-0009-5

**Published:** 2015-09-01

**Authors:** Takanori Nakamura, Yumi Ishida, Kasumi Ainai, Shigeto Nakamura, Satoru Shirata, Kazuhiro Murayama, Shin-ichiro Kurimoto, Katsuyasu Saigo, Ryo Murashige, Shuji Tsuda, Yu F. Sasaki

**Affiliations:** Department of Pharmaceutical Health Care, Faculty of Pharmaceutical Sciences, Himeji Dokkyo University, Hyogo, Japan; Faculty of Chemical and Biological Engineering, Hachinohe National College of Technology, Aomori, Japan; Department of Mechanical Engineering, Hachinohe National College of Technology, Aomori, Japan; Iwate Institute of Environmental Health Sciences, Iwate, Japan; Laboratory of Genotoxicity, Faculty of Chemical and Biological Engineering, Hachinohe National College of Technology, Tamonoki Uwanotai 16-1, Hachinohe, Aomori, 039-1192 Japan

**Keywords:** Anti-genotoxic effect, Micronuclei, Cigarette smoke, Antioxidant, *Connarus ruber*

## Abstract

**Introduction:**

According to published information, it has not been determined whether the inhalation of cigarette smoke can induce chromosome aberrations and/or point mutations in mice, though cigarette smoke is clearly carcinogenic to mice. We tested clastogenicity of inhaled cigarette smoke in mouse by a micronucleus test using peripheral erythrocytes. Since it is important to determine the in vivo anti-genotoxic effect against inhaled cigarette smoke to reduce the risk of tobacco carcinogenesis, we also tested in vivo anti-gnotoxic effect against inhaled cigarette smoke of a Connarus extract whose in vitro anti-genotoxic effect was shown.

**Results:**

Male ICR mice were exposed for 1 min to a 6-fold dilution of the smoke once a day for up to 14 consecutive days. Although the frequencies of reticulocytes with micronucleus (MNRETs) and erythrocytes with micronuclei (MN erythrocytes) did not increase within 72 h after a single inhalation of cigarette smoke, the frequency of MN erythrocytes increased significantly upon inhalation for 7 and 14 days. When the Connarus extract was fed to mice at >23.7 ppm during the inhalation period of 14 days, frequency of MN erythrocytes was significantly lower than that at 0 ppm. In vitro antioxidant activity of Connarus extract was almost same to that of vitamin C. The antioxidant activity of the Connarus extract might play an important role in its anti-genotoxic effect against cigarette smoke in vivo, like vitamins C.

**Conclusions:**

Consecutive inhalation of cigarette smoke is clastogenic to mouse bone marrow as shown by the increased frequency of MN erythrocytes. Also, it was shown the possibility that the Connarus extract reduces the risk of tobacco carcinogenesis.

## Introduction

Inhaled cigarette smoke has been shown to induce DNA single-strand breaks (SSB) in the known target organ (lung) and possible target organs (stomach and liver) of mice [1]. When inhaled, the tobacco smoke-derived nitrosamines N-nitrosodimethylamine (NDMA) and 4-(N-methyl-N-nitrosoamino)-1-(3-pyridyl)-1-butanone (NNK) are genotoxic in rat liver [2], and inhaled cigarette smoke induces the formation of DNA adducts in the lung [3, 4] and nasal mucosa of the rat [4]. However, it has not been determined whether cigarette smoke-induced DNA SSB and/or DNA adducts can develop into chromosome aberrations and/or point mutations in mice. To reduce the risk of tobacco carcinogenesis, it would be important to determine the in vivo anti-genotoxic effect of a new agent against inhaled cigarette smoke.

*Connarus ruber* Planchon, which is a dicotyledon of *Connaraceae*, a group that is distributed throughout tropical regions, grows in Maués in the Amazon. Extract of *Connarus ruber* has been studied as a potential therapeutic agent in the management of diabetes and related complications [5, 6]. We have shown that an aqueous extract of *Connarus ruber* cortex has genotoxicity-suppressing effect against UV in cultured human cells and suggested that its anti-genotoxic potential is due to an enhanced incision step of global genome repair (GGR) subpathways in nucleotide excision repair [7]. In addition, its anti-genotoxic effect has been examined in mice using a micronucleus assay. When mice received ≤2000 mg/kg of the *Connarus* extract by oral gavage at the same time as intraperitoneal injection of mitomycin C, a decrease in the frequency of micronucleated reticulocytes (MNRETs) was observed [7]. In this study, we investigate whether cigarette smoke-induced DNA damage can develop into chromosome aberrations and whether *Connarus* extract can show a genotoxicity-suppressing effect against inhaled cigarette smoke.

## Materials and methods

### Preparation of *Connarus* extract

A *Connarus* extract for feeding to mice was prepared like as for human consumption as follows. Four grams of *Connarus* cortex was put into 1000 mL of distilled water and boiled until the volume became 800 mL; then, the extract solution was separated from the cortex by filtration. The extract solution was evaporated and weighed. Based on the weight of evaporated extract, original concentration of evaporate extract in the extract solution was 758 ppm and the extract solution was diluted by 6 serial two-fold dilutions from 758 to 11.8 ppm using tap water for feeding to mice.

### Animals

Male ICR mice were obtained from SLC Japan, Inc. (Shizuoka), at 7 weeks of age and used for the inhalation study after an acclimatization period of 1 week. Four mice were randomly assigned to each *Connarus*-treated group. They were fed the commercial pellets MF (Oriental Yeast Industries, Tokyo) and *Connarus* extract ad libitum throughout the inhalation period. Mice in the control group were fed tap water. The animal room was 22 ± 2 °C with a 12-h light–dark cycle; the humidity was 30–50 %.

All procedures were approved by the Animal Research Committee, Faculty of Pharmaceutical Sciences, Himeji Dokkyo University.

### Cigarette smoke exposure

Four mice (combined body weight, ca. 160 g) were put into an 1800 mL polypropylene whole body inhalation chamber with 4 inlet pores at the top and an exhaust pore at the side.

Smoke of 35 mL was generated for duration of 2 s per puff of each cigarette according to the standard ISO method [3, 8] using a 50 mL glass syringe equipped with a cigarette holder for an unfiltered commercial cigarette (Piece, Japan Tobacco, Tokyo; containing 15 mg of nicotine and 1.3 mg of tar, according to the manufacturer). For an exposure we used eight cigarettes and eight syringes.

A 140 mL volume of the smoke from four cigarettes at a time was rapidly introduced into the chamber twice in turn without a break from the four inlet pores (the total smoke volume of 280 mL from eight cigarettes using eight syringes), and the pores were then closed immediately. According to the ISO standard [8] of 1 min interval, the mice were removed from the chamber and returned to their original cage after 1 min exposure. The fold dilution of the smoke by the air was calculated using the following equation: fold dilution = (1800–160)/280. The smoke concentration (5.85-fold dilution) was slightly higher than that used in a previous long term inhalation study (8 fold dilution) [9]. The mice were exposed to cigarette smoke once a day up to an exposure period of 14 days. Mice in sham control groups also transferred to smoking chamber every day during the 2 weeks of inhalation period and exposed to air instead of cigarette smoke.

### Micronucleus test in mice

The mice were fed the *Connarus* extract at ≤758 ppm ad libitum throughout a cigarette smoke exposure period of ≤14 days. Slides were prepared according to Hayashi et al. [10]. Five microliters of peripheral blood was obtained from the tail immediately before and after cigarette smoke exposure periods of 7 and 14 days. In another experiment, 5 μl of peripheral blood was obtained from the tail 24, 48, and 72 h after a single inhalation of cigarette smoke. This peripheral blood was placed on an acridine orange-coated glass slide and covered with a cover slip. For each mouse exposed to a single inhalation of cigarette smoke, the number of micronucleated reticulocytes (MNRETs) among 2000 reticulocytes (RETs) was scored using a fluorescence microscope with a blue excitation filter and a yellow barrier filter. For each mouse exposed to consecutive inhalations of cigarette smoke for 7 and 14 days, the numbers of MNRETs among 2000 RETs and micronucleated erythrocytes (MN erythrocytes) among 2000 erythrocytes were scored. The effect of the *Connarus* treatment on the incidence of MNRETs and MN erythrocytes was analyzed statistically by Dunnett’s test.

### Antioxidant activity assay (DPPH assay)

Vitamin C as a positive control was dissolved in distilled water. Aliquots of 10 μL of vitamin C solution and the *Connarus* extract were incubated for 2 h in 260 μL of DPPH (1,1-diphenyl-2-picrylhydrazyl) reaction solution (160 μL of 0.16 % DPPH in ethanol and 100 μL of 0.1 M acetate buffer), the mixture was mixed well with 840 μL of xylene, and then the absorbance of the xylene layer at 510 nm was measured. The inhibition ratio was calculated using the formula below. AS is the absorbance of the sample and AC is the absorbance of the control, in which there are no antioxidant reagents [11, 12].

$$ \mathrm{Inhibition}\ \left(\mathrm{z}\right)=100\times \left(1-\mathrm{A}\mathrm{S}/\mathrm{AC}\right) $$

## Results

**Fig. 1:**
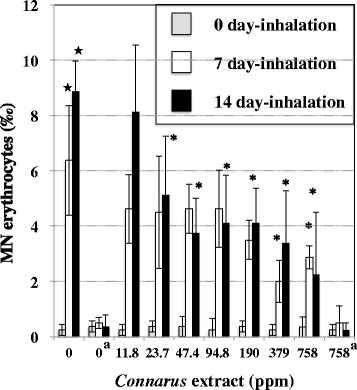
Effects of feeding on *Connarus* extracts on the frequency of MN erythrocytes in mice exposed to cigarette smoke for inhalation periods of 7 and 14 days. Mean ± SD of 4 mice are shown. *Significantly lower than *Connarus*-untreated control: *p* < 0.05. ★Significantly higher than 0 day-inhalation at 0 ppm *Connarus*: *p* < 0.05. ^a^Sham control group

Table 1 shows MNRET frequency in mice exposed to a single and consecutive inhalations to cigarette smoke. MNRET frequency did not increase within 72 h after a single inhalation. Also, after the inhalation period of 7 and 14 days, any significant differences were not observed in MNRET frequency between sham control and cigarette smoke inhaled groups. In contrast, as shown in Fig. 1, MN erythrocytes increased after inhalation periods of 7 and 14 days for 0 ppm *Connarus* extract. Within the inhalation period of 14 days, mice in sham control group fed *Connarus* extract at 0 ppm did not show a weight decrease and any clinical signs (Table 2). Also, cigarette smoke inhaled mice fed *Connarus* extract at 0 ppm did not show a weight decrease and any clinical signs showing the toxicity of the inhalation of cigarette smoke including cyanosis, sialorrhea, hypothermia, and hyperthermia. At all *Connarus* concentrations of ≤758 ppm, neither clinical signs showing the toxicity of the *Connarus* extract nor a weight decrease was observed within 14 days upon drinking *Connarus* extract (Table 2). The daily intake of water including *Connarus* extract was 5.63–7.50 mL/mouse, which was similar to the daily water intake in the control mice.

**Table 1:** MNRET frequency in the peripheral blood micronucleus test after a single and consecutive inhalation of cigarette smoke in mice that were not fed *Connarus* extracts

Exposure to smoke	MNRET (‰, mean ± SD)						
	0^a^	24^a^	48^a^	72^a^	0-day^b^	7-day^c^	14-day^d^
Single inhalation to cigarette smoke	1.75 ± 0.83	2.50 ± 0.50	2.25 ± 1.30	1.75 ± 0.43	-	-	-
Consecutive inhalation (Sham control)	-	-	-	-	1.50 ± 0.41	1.75 ± 0.45	1.50 ± 1.08
Consecutive inhalation to cigarette smoke	-	-	-	-	1.25 ± 1.10	2.00 ± 1.48	1.50 ± 0.83

^a^Sampling time after a single inhalation (h)

^b^Blood was sampled immediately before exposure to cigarette smoke

^c^Blood was sampled immediately after exposure period of 7 days

^d^Blood was sampled immediately after exposure period of 14 days

**Table 2:** Body weight in the mice during the period of exposure to cigarette smoke

*Connarus* extract (ppm)	Mortality within exposure period	Water or *Connarus* intake (mL/mouse/day)	Body weight (g, mean ± SD)		
			Period of exposure to cigarette smoke (day)		
			0	7	14
0^a^	0/4	5.56	37.3 ± 0.83	38.3 ± 1.79	38.0 ± 1.41
0^b^	0/4	6.25	36.8 ± 1.79	37.8 ± 2.49	38.3 ± 1.79
11.8^b^	0/4	7.50	37.8 ± 1.30	37.8 ± 1.30	37.8 ± 1.30
23.7^b^	0/4	6.25	37.3 ± 1.30	38.5 ± 1.66	38.5 ± 1.80
47.4^b^	0/4	7.50	36.8 ± 1.10	37.5 ± 1.11	39.0 ± 1.22
94.8^b^	0/4	6.25	37.8 ± 1.48	36.5 ± 1.12	37.8 ± 1.30
190^b^	0/4	5.63	35.3 ± 1.63	37.8 ± 2.49	35.0 ± 1.58
379^b^	0/4	6.25	35.5 ± 2.06	37.5 ± 2.69	35.5 ± 2.06
758^b^	0/4	6.25	34.5 ± 1.50	37.8 ± 2.48	35.3 ± 1.09
758^a^	0/4	6.61	35.3 ± 2.27	36.3 ± 0.83	36.5 ± 1.12

No clinical signs showing the toxicity of *Connarus* extract were observed

^a^Sham control group

^b^Mice were exposed for 1 min to a 5.85-fold dilution of the smoke once a day for up to 14 consecutive days

As shown in Fig. 1, MN erythrocyte frequencies did not increase in sham control mice fed 758 ppm *Connarus* extract, showing that the *Connarus* extract is not clastogenic to mouse bone marrow cells under the test condition. When the *Connarus* extract was fed to smoke-inhaled mice for the inhalation period of 14 days, the frequency of MN erythrocytes at ≥23.7 ppm was significantly lower than that at 0 ppm. When the *Connarus* extract at ≥379 ppm was fed to smoke-inhaled mice for the inhalation period of cigarette smoke of 7 days, the frequency of MN erythrocytes was significantly lower than that at 0 ppm.

**Fig. 2:**
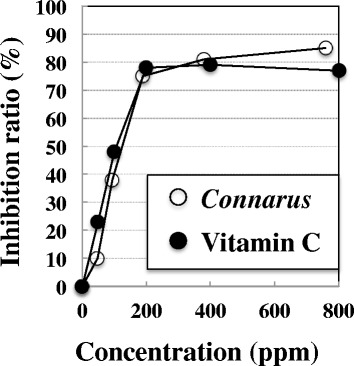
Antioxidant effects of *Connarus* extract. The ordinate axis indicates the ratio of inhibition of DPPH radicals. Each *symbol* represents the mean value of two experiments

Figure 2 shows the results of the antioxidant activities. Vitamin C as a positive control and the *Connarus* extract dose-dependently removed DPPH radicals. At the maximum concentration of vitamin C (800 ppm) and the *Connarus* extract (758 ppm), the inhibition ratio of DPPH radicals was 85 and 77 %, respectively, showing that in vitro antioxidant activity of *Connarus* extract is almost same to that of vitamin C.

## Discussion

Although the inhalation of cigarette smoke is clearly carcinogenic to mice [13], to our knowledge, few studies have shown its genotoxicity in mice, except for our previous study using the comet assay, in which a single inhalation of cigarette smoke caused DNA damage in the lung, stomach, and liver, but not in the kidney, brain, or bone marrow of mice [1]. The issue of whether DNA damage is repaired or persists is important for the fate of organs targeted by chemical carcinogens [14], but the issue of whether DNA damage induced by the inhalation of cigarette smoke develops into chromosome aberrations and/or gene mutations has not been studied. The induction of MNRETs was not observed within 72 h after a single inhalation of cigarette smoke, which coincides with our previous results that a single inhalation of cigarette smoke led to a negative comet response in mouse bone marrow [1]. On the other hand, the induction of MN erythrocytes was observed after 7 days’ exposure of mice to cigarette smoke, namely, 7 inhalations of cigarette smoke. It has been reported that hypothermia induces micronuclei in mouse bone marrow cells [15]. In this study, we did not detect any changes in body temperature in all mice by the palpation conducted every day during the inhalation period. Furthermore, any differences in general condition were not observed between mice in sham control and inhaled groups. Therefore, observed induction of MN erythrocytes in mice inhaled to cigarette smoke for ≥7 days could not be due to hypothermia.

MNRETs did not increase even after 7 and 14 consecutive inhalations to cigarette smoke. Therefore, it could not be considered that the accumulation of some genotoxic factors in mouse body results in the induction of MN erythrocytes after 7 and 14 consecutive inhalations. In humans and many other mammals, MN erythrocytes are removed rapidly by the spleen. In mice, however, it has been well known that MN erythrocytes are not removed [16]. Considering that reticulocytes are immature erythrocytes and develop into mature erythrocytes within 72 h, MNRETs can reflect only last 1 and 2 inhalations even after 7 and 14 consecutive inhalations. Therefore, it would be considered that the induction of MNRETs by one inhalation of cigarette smoke is too small to be detected, but that the induced MN erythrocytes accumulate to a level that is sufficiently high to be detected after 7 inhalations of cigarette smoke. While cigarette smoke-induced DNA adduct formation via metabolic activation of aromatic carcinogens is considered to be a determinant of tobacco carcinogenesis, free radicals may also play an important role [17, 18]. High concentrations of free radicals are present in both the gas and the particulate (tar) phases of cigarette smoke. The former contains small carbon- and oxygen-centered free radicals, such as peroxyl radicals, and the latter a hydroquinone-semiquinone-quinone redox system, which elicits the formation of hydroxyl radicals via hydrogen peroxide [18, 19]. The gas phase also contains carbonyl sulfide, which produces hydroxyl radicals from hydrogen peroxide [17]. The highly reactive hydroxyl radicals are involved in the formation of DNA damage in vitro [19] and 8-hydroxydeoxyguanosine in vitro [20], as well as in smokers [21, 22].

Factors producing protective effects against the genotoxicity of cigarette smoke in vivo have not been well reported. Our previous study showed that antioxidants, such as vitamins C, exhibited protective effects against the genotoxicity of cigarette smoke in vivo and suggested that free radicals were a source of the damage [1]. In this study, aqueous extract of *Connarus ruber* cortex was also shown to have antioxidant activity and in vitro antioxidant activity of *Connarus* extract is almost same to that of vitamin C. Therefore, we could speculate one possibility that the antioxidant activity of the *Connarus* extract plays an important role in its anti-genotoxic effect against the clastogenicity of cigarette smoke in vivo, like vitamin C.

The anti-genotoxic effect of the *Connarus* extract was studied in cultured human cells and it was shown to have a suppressive effect against the induction of micronuclei by methyl nitrosourea, mitomycin C (MMC), or ultraviolet C [7]. In addition, it was shown to suppress the induction of MNRET by MMC in mice [7]. The genotoxicity-suppressing effect was further studied by comet assay; it was suggested that the anti-genotoxic potential is due to an enhanced incision step of global genome repair (GGR) subpathways in nucleotide excision repair and that a number of anti-genotoxic components with different modes of anti-genotoxicity are contained in *Connarus* extract [7]. Cigarette smoke contains more than 5000 chemicals; their interactions may be additive, synergistic, or inhibitory [23]. Thus, a number of anti-genotoxic modes with different types of anti-genotoxicity other than the antioxidant one should play roles in the exertion of in vivo anti-genotoxic effect of *Connarus* extract against inhaled cigarette smoke.

## Conclusion

In conclusion, it was shown the possibility that the *Connarus* extract reduces the risk of tobacco carcinogenesis.
